# Novel MDM2 inhibitor SAR405838 (MI-773) induces p53-mediated apoptosis in neuroblastoma

**DOI:** 10.18632/oncotarget.12634

**Published:** 2016-10-13

**Authors:** Jiaxiong Lu, Shan Guan, Yanling Zhao, Yang Yu, Yongfeng Wang, Yonghua Shi, Xinfang Mao, Kristine L. Yang, Wenjing Sun, Xin Xu, Joanna S. Yi, Tianshu Yang, Jianhua Yang, Jed G. Nuchtern

**Affiliations:** ^1^ Department of Ophthalmology, Shanghai Tenth People's Hospital, Tongji University School of Medicine, Shanghai 200072, China; ^2^ Texas Children's Cancer Center, Department of Pediatrics, Dan L. Duncan Cancer Center, Baylor College of Medicine, Houston, TX 77030, USA; ^3^ Division of Pediatric Surgery, Michael E. DeBakey Department of Pediatric Surgery, Dan L. Duncan Cancer Center, Baylor College of Medicine, Houston, Texas 77030, USA; ^4^ Department of Pathology, University of Texas MD Anderson Cancer Center, Houston, TX 77030, USA; ^5^ Department of Pathology, Basic Medicine College of Xinjiang Medical University, Urumqi, Xinjiang 830011, China; ^6^ Xinjiang Key Laboratory of Biological Resources and Genetic Engineering, College of Life Science and Technology, Xinjiang University, Urumqi, Xinjiang 830046, China

**Keywords:** neuroblastoma, MDM2 inhibitor, SAR405838, chemo-resistance, chemotherapy

## Abstract

Neuroblastoma (NB), which accounts for about 15% of cancer-related mortality in children, is the most common childhood extracranial malignant tumor. In NB, somatic mutations of the tumor suppressor, p53, are exceedingly rare. Unlike in adult tumors, the majority of p53 downstream functions are still intact in NB cells with wild-type p53. Thus, restoring p53 function by blocking its interaction with p53 suppressors such as MDM2 is a viable therapeutic strategy for NB treatment. Herein, we show that MDM2 inhibitor SAR405838 is a potent therapeutic drug for NB. SAR405838 caused significantly decreased cell viability of p53 wild-type NB cells and induced p53-mediated apoptosis, as well as augmenting the cytotoxic effects of doxorubicin (Dox). In an *in vivo* orthotopic NB mouse model, SAR405838 induced apoptosis in NB tumor cells. In summary, our data strongly suggest that MDM2-specific inhibitors like SAR405838 may serve not only as a stand-alone therapy, but also as an effective adjunct to current chemotherapeutic regimens for treating NB with an intact MDM2-p53 axis.

## INTRODUCTION

Arising from the sympathetic nervous system, neuroblastoma (NB) is an embryonic tumor that accounts for about 15% of cancer-related deaths in children [1, 2]. Despite encouraging progress in the outcome during the past years, the overall survival rate for high-risk NB patients remains dismal. Current therapies for high-risk NB, with frequent recurrence and formidable chemo-resistance, are still insufficient. Thus, novel targeted therapies for high-risk NB are urgently needed.

p53 is a major tumor suppressor that induces cell growth arrest, apoptosis, and senescence, along with being one of the most important regulators of multiple signaling pathways [3–5]. According to previous studies, p53 is mutated in over 50% of all cancers. However, p53 mutations are exceedingly rare in NB, with less than 2% occurrence in primary tumors [6, 7]. p53 is reported to irreversibly induce apoptotic responses in NB cells once stabilized [8–15], suggesting that the stabilization of p53 is a possible therapeutic strategy for NB treatment. The cellular expression, function, and stabilization of p53 are governed by a complex regulatory network. Of the factors that regulate p53 activities, Murine double minute 2 homolog (MDM2) is one of the major negative regulators [16]. MDM2 binds to p53 and mediates its poly-ubiquitination and degradation, thus, inhibiting p53 tumor suppressor function [2, 7, 16–23].

In NB cells, MYCN down-regulates p53 and up-regulates MDM2 at the transcriptional level [24, 25]. PPM1D inhibits p53 tumor suppressor function by dephosphorylating p53 at Ser15 [26, 27]. UBE2N promotes the formation of monomeric p53 that results in cytoplasmic translocation and subsequent loss of tumor suppressor function [20]. DUSP26 inhibits p53 tumor suppressor function by dephosphorylating p53 at Ser37 and Ser46 [28, 29]. USP7, an MDM2 deubiquitinase, deubiquitinates MDM2 and prolongs its half-life to inhibit the p53 tumor suppressor function [21]. The overexpression of MDM2 is a typical example of non-mutational p53 inactivation in NB cells, indicating that the inhibition of p53-MDM2 interactions is capable of restoring p53 tumor suppressor function. p53-MDM2 binding antagonists are a novel class of anti-tumor therapeutics in malignancies with intact p53 function, and has been proposed to be a potential strategy for NB therapy [9].

Several MDM2 inhibitors, such as Nutlin-3a, MI-219, MI-63, RG7388, RITA and MLN-8237, etc., have all been evaluated previously. However, of the well-studied MDM2 inhibitors, Nutlin-3a may result in acquired chemo-resistance, and MI-63 fails to demonstrate *in vivo* efficacy. Additionally, many drugs are restricted from clinical applications due to poor absorption, toxicity to normal tissues, and the development of resistance [30–36]. Thus, an ideal MDM2 inhibitor should have both efficient antitumor activity and minimal/improved toxicity.

SAR405838 (MI-773), currently in phase-I clinical trials, is a novel, potent, and orally available MDM2 antagonist that blocks the interaction between MDM2 and p53. It showed significant antitumor effects by stabilizing p53 function. Moreover, SAR405838 is effective in liposarcoma, lymphoma, and leukemia with negligible toxicity in animal xenograft models [30, 37, 38]. In this paper, we evaluate the effects of SAR405838 on NB cell lines. Our results demonstrated that SAR405838 induces p53-mediated apoptosis in NB, suggesting that this inhibitor is a potential therapeutic tool to add to the armamentarium for NB patients.

## RESULTS

### MDM2 inhibitor SAR405838 suppresses cell proliferation in the p53 WT NB cell lines

**Figure 1:**
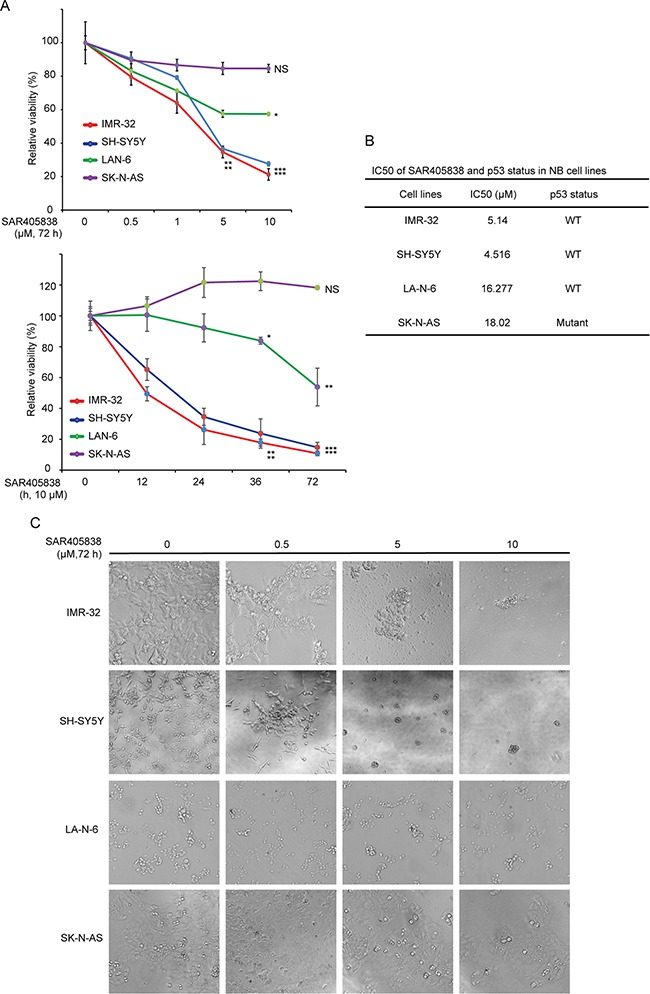
SAR405838 shows cytotoxic effects on p53 wild-type NB cell lines **A.** Four NB cell lines were treated with increasing concentrations of SAR405838 for 72 hours or increasing treatment hours at 10 μM. Cell viability was assessed by CCK-8 assay. Data is represented as % vehicle ± S.D. with P < 0.05 (*), P < 0.01 (**), or P < 0.001 (***) (Student's t-test, two-tailed) as indicated. **B.** The IC50 values of SAR405838 in each cell line listed were calculated in Prism 5 and based on the data collected in the cell viability assay. p53 status in NB cell lines was also shown. **C.** Morphological changes of the four different NB cell lines treated with increasing concentrations of SAR405838 for 72 hours were shown.

To determine the antitumor effect of SAR405838, the CCK-8 assay was used to test whether SAR405838 could affect cell proliferation in a panel of NB cell lines. In total, we selected one p53 mutant (SK-N-AS) and three p53 wild-type (SH-SY5Y, IMR-32, and LA-N-6) cell lines. The cell viabilities of SY5Y and IMR-32 were greatly reduced both in a dose-dependent manner with increasing concentrations of SAR405838 and in a time-dependent manner with increasing treatment time (Figure 1A). This effect was attenuated in LA-N-6 due to its innate chemo-resistance; however, when compared to a lack of SAR405838 treatment, differences were still observed. In contrast, the p53 mutant cell line, SK-N-AS, exhibited no reduced cell viability with SAR405838 treatment (Figure 1A, 1C). The IC50 of SAR405838 in all four cells lines was calculated (Figure 1B), and our results indicate that SAR405838 inhibits cell proliferation in a dose-dependent manner in NB p53 WT cell lines, but not in p53 mutant lines. These results were validated by the flow cytometry that SAR405838 promoted apoptosis in p53 WT cell line IMR-32, but not in the p53 mutant cell line SK-N-AS (Supplementary Figure S1).

### MDM2 inhibitor SAR405838 inhibits colony formation ability of the p53 WT NB cell lines

**Figure 2:**
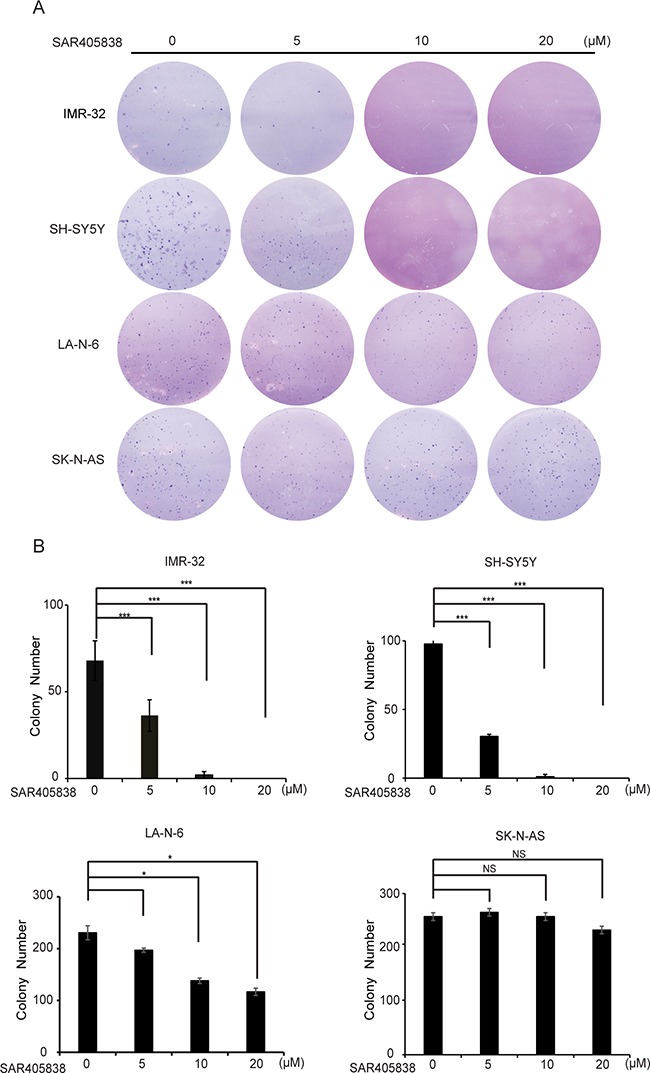
SAR405838 suppresses anchorage-independent growth of NB cells **A.** A panel of four NB cell lines were seeded in six-well plates with indicated concentrations of SAR405838 and agar, and grown for 2 to 3 weeks. Cells were stained with crystal violet for 4 hours, and images were obtained. **B.** Colonies were counted and colony numbers were represented as % vehicle ± S.D. with P < 0.05 (*), P < 0.01 (**) or P < 0.001 (***) (Student's t-test, two-tailed) as indicated.

To evaluate whether SAR405838 could inhibit the colony formation abilities of NB cell lines, we performed soft agar assays. In this assay, we found that the p53 WT cell lines (SH-SY5Y, IMR-32, and LA-N-6), but not the p53 mutant ones (SK-N-AS), showed a significantly decreased ability to form colonies after SAR405838 treatment compared with vehicle-treated control (Figure 2A). Colony numbers were calculated in each group (Figure 2B), revealing that SAR405838 significantly attenuated anchorage-independent growth of the p53 WT NB cells in a dose-dependent manner.

### SAR405838 induces p53-mediated apoptosis in p53 WT NB cell lines

**Figure 3:**
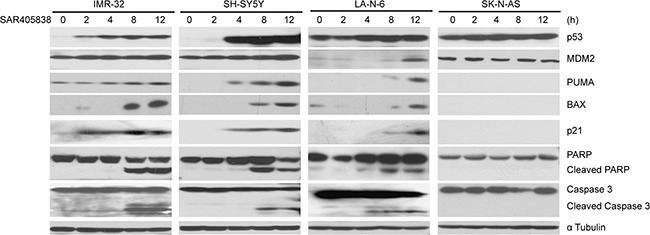
SAR405838 activates p53 downstream signaling pathway, and induces apoptosis in p53 WT NB cells IMR-32, SH-SY5Y and SK-N-AS cells treated with 20 μM of SAR405838 and LA-N-6 cells with 50 μM of SAR405838 for various time points (0-12 hours), were lysed and subjected to SDS-PAGE followed by immunoblotting with the antibodies indicated. The anti-α Tubulin antibodies were used as a loading control for whole cell extracts.

According to prior studies, SAR405838 inhibits MDM2 from ubiquitinating and binding to p53, consequently, stabilizing it [39]. As a result, we hypothesized that SAR405838 could block the p53/MDM2 axis and promote activation of the p53 pathway in the p53 WT NB cells. The level of p53 and downstream p21, BAX, PUMA, as well as MDM2 and the apoptosis related protein PARP and Caspase 3, were examined by immunoblotting assays. Consistent with our hypothesis, SAR405838 induced p53 accumulation in all p53 WT NB cell lines (Figure 3A), whereas the p53 level in SK-N-AS cells was not affected (Figure 3B). Moreover, in strong contrast to the control (0 h), all the cell lines except SK-N-AS demonstrated obvious PARP and Caspase 3 cleavage with increasing treatment time. Our data indicates that SAR405838 promotes p53-mediated apoptosis in the p53 WT NB cells.

### SAR405838 enhances the cytotoxic effect of dox in the p53 WT NB cell lines

**Figure 4:**
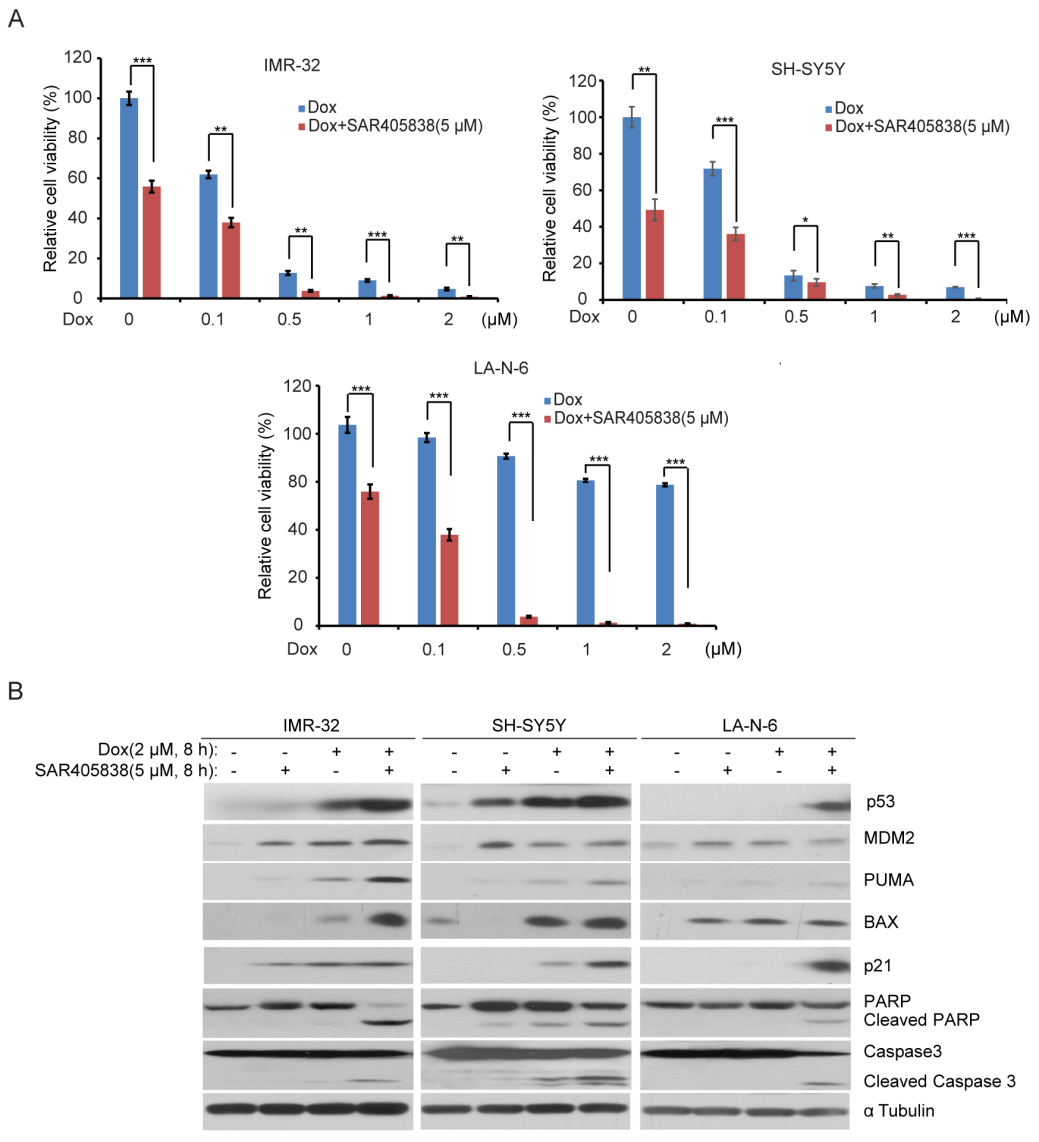
SAR405838 enhances the cytotoxic effect of Doxorubicin on NB cell lines **A.** IMR-32, SH-SY5Y, and LA-N-6 cells were seeded in 96-well plates and incubated with the indicated concentrations of Dox plus DMSO or 5 μM of SAR405838 for 24 hours. Cell viability was then measured by CCK-8 assay. **B.** IMR-32, SH-SY5Y, and LA-N-6 cells were treated with either Dox (2 μM) alone, SAR405838 (5 μM) alone, or a combination of both for 8 hours. Then cells were lysed and subjected to SDS-PAGE followed by immunoblotting with the antibodies indicated. The α Tubulin antibodies were used as a loading control for whole cell extracts.

In the treatment of high-risk NB, the effects of monotherapies are less effective due to the development of chemo-resistance [40]. Since most cancers rapidly acquire chemo-resistance, the best strategies are combination therapies. We evaluated the combination effects of SAR405838 and the traditional anti-NB chemotherapy drug doxorubicin (Dox) via a panel of three p53 WT NB cell lines: IMR-32, SH-SY5Y, and LA-N-6. In the chemo-sensitive cell lines (IMR-32 and SH-SY5Y) we observed that SAR405838 enhanced Dox-induced p53, MDM2, p21, BAX and PUMA expressions, and augmented Dox-induced PARP and Caspase 3 cleavage compared to the effects of Dox alone (Figure 4A, 4B). Then, to test whether SAR405838 could overcome established chemo-resistance in NB cells, the chemo-resistant LA-N-6 cell line was treated with varying doses of Dox with or without SAR405838. Remarkably, the addition of SAR405838 augmented Dox-induced cell apoptosis in LA-N-6 cells, suggesting that SAR405838 sensitized LA-N-6 cells to Dox-induced apoptosis. Together, these results indicate that the addition of SAR405838 greatly augmented Dox-induced apoptosis in all p53 WT NB cell lines, suggesting that the combination of SAR405838 with Dox not only induces stronger apoptosis in the p53 WT NB cells, but also effectively combats established chemo-resistance compared to single drug treatment.

### SAR405838 induces apoptosis in the p53 WT NB tumor cells of the xenograft mouse model

**Figure 5:**
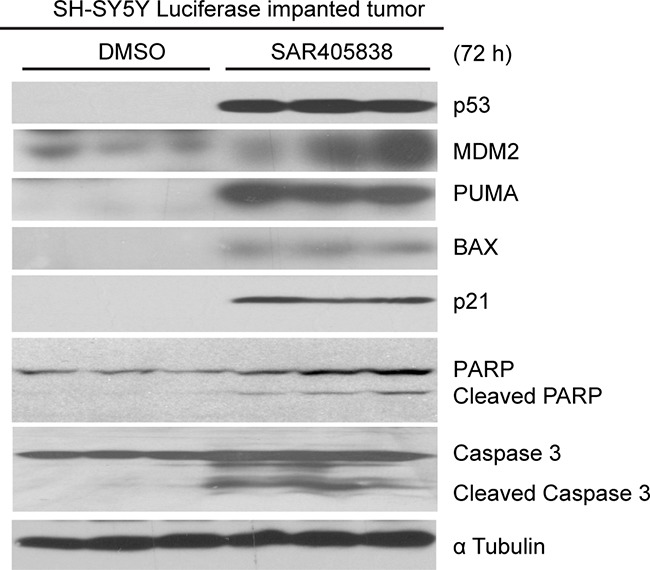
SAR405838 induces p53-mediated apoptosis in tumor cells in mouse model of NB The nude mice bearing tumors were treated with DMSO or 30 mg/kg of SAR405838 (intraperitoneal injection) daily for 72 hours. The tumors were then harvested and subjected to SDS-PAGE followed by immunoblotting with the antibodies indicated.

An orthotopic NB xenograft mouse model was utilized to test whether SAR405838 could induce apoptosis *in vivo*. Luciferase-transduced SH-SY5Y cells were surgically injected into the left renal capsule of nude mice. Two weeks after injection, tumor signals were detected by bioluminescent imaging (data not shown). Mice were randomly divided into two groups and treated with either dimethylsulfoxide (DMSO) (carrier control) or SAR405838 (intraperitoneally injected 30 mg/kg daily for 3 days). Then, tumors from the mice treated with SAR405838 were harvested and analyzed for p53 pathway and apoptotic effectors. Consistent with the *in vitro* data, SAR405838 induced p53, MDM2, p21, BAX and PUMA expression, as well as PARP and Caspase 3 cleavage in NB tumor cells of the xenograft mouse model (Figure 5). These results demonstrate that SAR405838 can effectively induce p53-mediated apoptosis in the p53 WT NB tumors developed in the xenograft mouse model.

### Intact p53-MDM2 axis is required for SAR405838 to induce p53 target gene expression in NB cells

**Figure 6:**
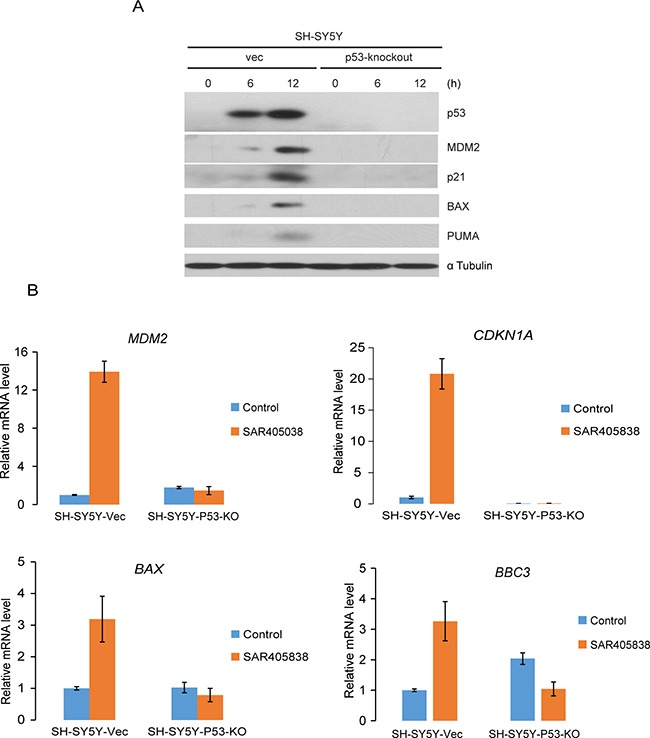
Intact p53-MDM2 axis is required for the effect of SAR405838 on NB cells **A.** SH-SY5Y-vector and SH-SY5Y-p53-knockout cells were treated with10 μM SAR405838 for 0, 6, 12 hours, respectively. The cells were then lysed and subjected to SDS-PAGE, followed by immunoblotting with immunoblotting with the antibodies indicated. The α-Tubulin antibodies were used as a loading control for whole cell extracts. **B.** SH-SY5Y-vector and SH-SY5Y-p53-knockout cells were treated with 10 μM SAR405838 for 0 or 12 hours, respectively and collected for total RNA extraction. The relative RNA level for each gene indicated was measured by RT-PCR analysis.

To determine whether SAR405838 inhibits cell proliferation and induces apoptosis in a p53 dependent manner, we generated a p53-knockout SH-SY5Y cell line using CRISPR-Cas9-mediated genomic editing technology (Supplementary Figure S2). We found that p53 knockout blocked the ability of SAR405838 to induce p53 target gene expression at both the protein (Figure 6A) and transcription (Figure 6B) level. These results suggest that SAR405838 causes p53 activation in NB cells.

### The comparison effect of three MDM2 antagonists on NB cell line IMR-32

RG7388 and Nutlin-3 are well studied MDM2 small-molecular inhibitors that have been reported to potently stabilize p53 in p53 WT NB cell lines [22, 41]. We compared the inhibitory effect of SAR405838 with RG7388 and Nutlin-3 on one p53 wild-type NB cell line, IMR-32.

**Figure 7:**
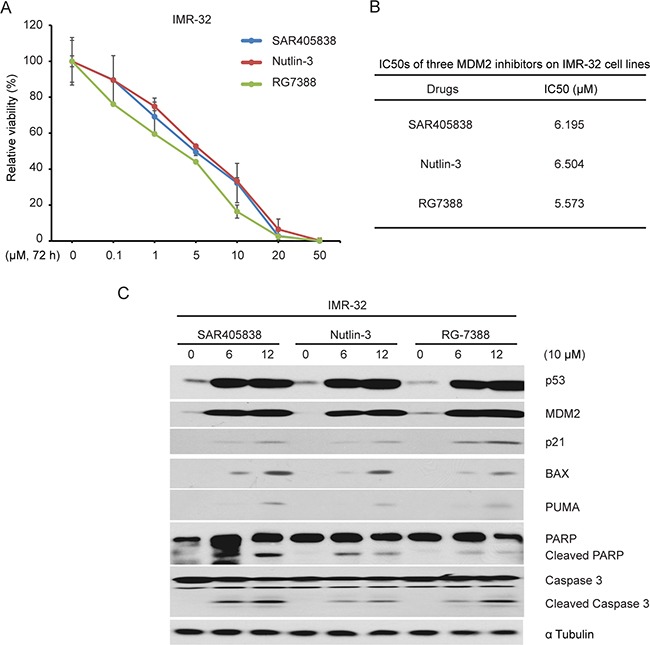
The effect of three MDM2 antagonists SAR405838, RG7388 and Nutlin-3 on the proliferation of a p53 WT NB cell line **A.** IMR-32 cells were treated with the increasing concentrations of SAR405838, Nutlin-3 and RG7388 for 72 hours, respectively. Cell viability was then measured by CCK-8 assay. **B.** The IC50s of SAR405838, Nutlin-3 and RG7388 in IMR-32 cell line were calculated based on the data collected in the cell viability assay by using Prism 5. **C.** The IMR-32 cells were treated with an identical 10 μM concentrations of SAR405838, Nutlin-3 and RG7388 for 12 hours. Cells were then lysed and subjected to SDS-PAGE followed by immunoblotting for the antibodies indicated. The α Tubulin antibodies were used as a loading control for whole cell extracts.

We found that these three compounds had similar inhibitory effect on the cell proliferation (Figure 7A) and p53 target gene expression as well as PARP and Caspase 3 cleavage (Figure 7B). These data suggest that SAR405838 has similar efficacy as RG7388 and Nutlin-3 *in vitro*.

## DISCUSSION

p53, regulated by multilateral upstream signals, is a crucial cellular tumor suppressor [14, 42, 43]. A strategy to restore p53 activity could be a promising therapeutic option for NB since more than 98% of p53 downstream functions are still intact in NB [44]. MDM2 is the major negative regulator of p53 tumor suppressor. Recently, small molecule inhibitors interfering with the p53/MDM2 axis are generating intense interest. SAR405838 is the latest oral MDM2 antagonist that attracted our attention because it has demonstrated high efficacy in multiple cancer models. SAR405838 effectively stabilizes p53 and activates the p53 pathway, resulting in abrogated cellular proliferation, cell-cycle arrest, and apoptosis in liposarcoma, lymphoma and leukemia with an intact MDM2-p53 axis [30, 37, 38]. The chemical structure of SAR405838 not only mimics three key amino acid residues of p53, but also induces refolding of the short, unstructured MDM2 N-terminus to achieve its improved affinity and efficacy [30, 38]. In addition, compared to already available intraperitoneally injected MDM2 inhibitors, SAR405838 should have better absorption and a higher potential for clinical applications since it is orally available. In addition, prior studies suggest that SAR405838 is more potent than relevant MDM2 inhibitors such as Nutlin3a and MI-63, since under identical conditions, lower SAR405838 concentrations are needed to produce the same effect compared with those of Nutlin3a and MI-63 [35]. In addition, when compared with other MDM2 antagonists RG7388 and Nutlin-3, which have demonstrated significant efficacy in a variety of cancer types *in vitro* [45–47], SAR405838 had shown a similar inhibitory effect on NB cells.

With the collaborations of cooperative group trials in the US and around the world, low and intermediate risk NB have overall good outcomes. However, high-risk NB still demonstrates a need for marked improvement in outcomes, and novel, mechanism-based therapeutic trials could play an important role to bridge this gap. We investigated whether SAR405838 could be an effective agent and demonstrate prominent anti-proliferative effects and induction of apoptosis in p53 WT cell lines. Also, as high-risk NB is often associated with chemo-resistance, we were pleased to note that SAR405838 could sensitize chemo-resistant cell line LA-N-6 to Doxorubicin-induced apoptosis. These results suggest that SAR405838 could potentially be beneficially combined with existing treatment regimens for high-risk NB.

Here, we report the inhibitory effects of the MDM2 inhibitor SAR405838 (a potent p53 stabilizer) on NB cells. SAR405838 inhibits cell proliferation, induces apoptosis and upregulates mRNA and protein expression of the p53 pathway on p53 WT NB cell lines. Moreover, consistent with our *in vitro* data, SAR405838 is capable of inducing p53-mediated apoptosis in an orthotopic NB mouse model. While single treatment can not completely overcome the chemo-resistance in the NB cell line LA-N-6, the combination with Dox did lessen this concern, sensitizing NB cells to Dox-mediated cytotoxic effect.

In conclusion, our preclinical studies provide the rationale to evaluate MDM2 inhibitor SAR405838 in the therapeutic regimen of NB patients with intact MDM2-p53 axis.

## MATERIALS AND METHODS

### Antibodies and reagents

Anti-p53 antibodies (DO-1) (sc-126) and p21 antibodies (SX118) (sc-53870) were obtained from Santa Cruz Biotechnology (Santa Cruz Biotechnology, Dallas, TX, USA); Anti-PARP (9532 S), anti-Caspase-3 (9662S), anti-Mouse, (7076S) and anti-Rabbit (7074S) antibodies were from Cell Signaling Technology (Cell Signaling Technology, Danvers, MA, USA); and anti-α Tubulin (10D8) was obtained from Santa Cruz Technology (Santa Cruz Technology, Dallas, TX, USA). SAR405838 (S7649) and Nutlin-3 (S1061) was purchased from Selleckchem (Selleckchem, Houston, USA). RG7388 (A3763) was from APExBIO (APExBIO, Houston, TX, USA). Doxorubicin (Dox, D1515) was from Sigma (Sigma-Aldrich Corp, St. Louis, MO, USA).

### Cell lines and cell culture

The p53 wild-type human NB cell lines (IMR-32, LA-N-6 and SH-SY5Y) were cultured in RPMI Medium 1640 (RPMI) (Lonza, Walkersville, MD, USA), supplemented with 20% (v/v) heat-inactivated Fetal Bovine Serum (FBS) (SAFC Biosciences, Lenexa, KS, USA), 100 units/mL penicillin, and 100 μg/mL streptomycin. The p53 mutant cell line (SK-N-AS) was grown in RPMI containing 10% (v/v) heat-inactivated FBS, 100 units/mL penicillin, and 100 μg/mL streptomycin. All cells were maintained in a humidified incubator at a constant temperature of 37°C & 5% CO_2_. SH-SY5Y cell line with luciferase expression was generated with transduction of lentiviral luciferase virus containing a neo selection marker, and then selected with 800 μg/ml G418 (Enzo Life Sciences, Farmingdale, NY, USA) for 10 days. All experiments were performed with the prerequisite that cells were under exponential growth conditions.

### Cell viability assay

Cell viability was calculated using the Cell Counting Kit-8 (CCK-8, WST-8[2-(2-methoxy-4-nitrophenyl)-3-(4-nitrophenyl)-5-(2, 4-disulfophenyl)-2 H-tetrazolium, monosodium salt]) (Dojindo Laboratories, Rockville, MA, USA). Cells were plated and grown in 96-well clear-bottom plates starting at 1 × 10^4^ cells/well. After 24 hours of incubation, the medium was changed. Increasing concentrations of SAR405838, Dox, or a combination of both were added to the wells, and the cells were then incubated for another 24 hours or 72 hours. Then, a mixture of 10 μL of CCK-8 and 190 μL of RPMI with 10% or 20% FBS were added into each well respectively. After 1 hour of incubation, the absorbance was measured at 450 nm using a microplate reader. Each experiment was performed in duplicates of six and background reading of the medium was subtracted from each well to standardize the results.

### Cell imaging

A total of four NB cell lines were seeded in 96-well plates at appropriate concentrations. After 24 hours of treatment with indicated concentrations of SAR405838, cell morphologies were observed and captured using an optical microscope.

### Flow cytometry and propidium iodide (PI) staining assay

The experiment was performed as described previously [48]. Briefly, cells were seeded with 1 × 10^6^ cells/6 cm dish, After 24 h in culture, cells were treated with 10 μM of SAR405838 for 0 or 24 hours. Cells were trypsinized, resuspended in RPMI 1640 medium, and then centrifuged at 200 g for 3 min at 4°C. Cells were then washed with cold 1× PBS (add 1% BSA) twice, and resuspended at a density of 1 × 10^6^ cells/ml in 1× binding buffer (51-66121E; BD Biosciences, San Jose, CA, USA). Afterwards, 100 μl of non-fixed cell suspension was transferred into a new tube and stained with 5 μl of 50 μg/mL PI staining solution (51-66211E; BD Biosciences). The cells in the tubes were gently vortexed and incubated for 15 min at RT (25°C) in the dark. Unstained cells were used as a negative control, and untreated cells were used as a control for treated cells. Then flow cytometry analyses were performed on a LSR-II flow cytometer (BD Biosciences) using BD FACDiva software v.6.0.

### Colony formation assay

The soft agar assay for detecting colony formation abilities was performed as previously described [20]. Namely, a 5% (w/v) base agar layer was made by adding agar (214220, Difco Laboratories, Detroit, MI, USA) into distilled water and then autoclaving the mixture for 50 min before cooling in a 56°C water bath. This solution was then mixed with RPMI and 10% FBS to a final concentration of 0.5%. To make the bottom agar layer, 2 mL of the 0.5% agar/RPMI solution were added to each well and cooled until semi-solid. The top agar layer was made of 1.5 ml 0.3% agar, and each NB cell line was counted and added to the mixture at 1 × 10^4^ cells/well along with the indicated concentrations of SAR405838. Cells were grown at 37°C for 2 to 3 weeks, and subsequently stained with 500 μL of 5 mg/mL Thiazolyl Blue Tetrazolium Bromide (MTT, M5655, Sigma). Images were captured by the microscope, and colonies were counted after 4 hours. Each assay was performed in triplicate.

### Protein immunoblotting

Cells were lysed and then separated by SDS-PAGE. After each treatment, cells were washed twice with ice cold PBS and then lysed on a rotator at 4°C for 30 min in cooled RIPA buffer (150 mM NaCl, 50 mM Tris-HCl at pH 7.4, 50 mM sodium fluoride, 1 mM EDTA, 1 mM dithiothreitol, 1 mM phenylmethylsulfonyl fluoride, 1 mM benzamidine, 0.1 mM sodium orthovanadate, 10 μg/mL leupeptin, 1% NP-40, 0.25% sodium deoxycholate, and phosphatase inhibitor cocktail 2 and 3 (p5726 and p0044, Sigma)). We collected cell pellets after centrifuging for 5 min at 6,000 rpm and then lysed the cells on ice for 30 min followed by centrifuging for 15 min at 13,000 rpm to collect supernatants as cell lysates. Protein concentration in cell lysates were measured using Bradford reagent (Bio-Rad Laboratories, Hercules, CA, USA), and samples were mixed in a 3:1 ratio (v/v) with 4 × loading buffer, respectively, and heated at 100°C for 6 min. Lysates were separated by SDS-PAGE, transferred to PVDF (polyvinylidence fluoride) membranes (BioRad), blocked with 5% milk for one hour at room temperature (25°C), and probed with appropriate dilutions of indicated primary antibodies overnight at 4°C. The membranes were then incubated with anti-mouse or rabbit IgG conjugated with horseradish peroxidase at room temperature for 1hr. The ECL-Plus Western detection system (GE Health Care, Buckinghamshire, UK) was then used for chemiluminescent visualization.

### Antitumor efficacy in an orthotopic mouse model of NB

Four to six-week-old female athymic NCR nude mice were purchased from Taconic (Taconic, Hudson, NY, USA) and maintained under barrier conditions (pathogen-free conditions provided by plastic cages with sealed air filters). The preclinical mouse model of NB was established using orthotopic (intrarenal) implantation of the NB cells as described previously [49]. Briefly, a transverse incision was created over the left flank of the nude mouse and 1.5 × 10^6^ human luciferase-transduced SH-SY5Y cells in 0.1 ml of PBS were surgically injected into the left renal capsule and toward the superior pole of the left kidney of the nude mice.

After engrafting for 4 to 5 weeks, mice bearing tumors with similar sizes (using bioluminescent imaging to monitor tumor growth) were randomly divided into two groups and treated with either DMSO or SAR405838 (30 mg/kg, intraperitoneal injection once daily). Three days later, the mice were sacrificed and the tumors were harvested and lysed for immunoblotting. All mice were handled according to protocols approved by the Institutional Animal Care and Use Committee of the Baylor College of Medicine.

### Generation of the *TP53* gene knockout cell line through CRISPR-Cas9-mediated genomic editing technology

The lentiviral vector with p53 gRNA (CCTGCATGGGCGGCATGAAC) and Cas9-2A-puromycin expression cassettes was transfected with packaging vectors (Hgpm, Tat-1b, Rev-1b and VSVG) into 293T cells. After 24 hours of incubation, the viral supernatants were collected. The viral supernatants mixed with 4 μg/ml polybrene were used to transduce SH-SY5Y cells (2.5 × 10^6^ cells/dish). The transduced cells were then selected by treating cells with 0.5 μg/ml puromycin for 3 to 4 days. The single cell clone with p53 knockout was verified by PCR flanking the target site followed by cloning the PCR products and sequencing analysis.

### Quantitative reverse transcription-PCR

Total RNAs were extracted from cell lines using TRIzol LS Reagent (Invitrogen™ Ambion™). RNA purity and quantity were determined using a spectrophotometer measuring absorbance at 260/280 nm. The sequences for *TP53*, *MDM2*, *CDKN1A*, *BAX* and *BBC3* were retrieved from GenBank of National Center for Biotechnology Information (NCBI) database. Based on NCBI database, primers of *TP53*, *MDM2*, *CDKN1A*, *BAX* and *BBC3* were designed as 5′-CAGCACATGACGGAGGTTGT, 3′-TCATCCAAATACTCCACACGC; 5′-GAATCATCG GACTCAGGTACATC, 3′-TCTGTCTCACTAATTGCTC TCCT; 5′-CTGAAGGGTCCCCAGGTG, 3′-CAGGCTT CCTGTGGGCGG; 5′-CCCGAGAGGTCTTTTTCCG AG, 3′-CCAGCCCATGATGGTTCTGAT; and5'-TCAAC GCACA GTACGAGCGG, 3′-AGG CACC TAATTGGGCTCC; respectively. These sets of primers were synthesize by Sigma-ALDRICH. Then, primers and templates were mixed according to the SensiFAST SYBR Hi-ROX One-Step Kit (BIO-73005, BIOLINE). The mRNA level for each gene was detected by Applied Biosystems™ Real-Time PCR Instruments.

### Statistical analysis

All values were presented as mean ± standard deviation (SD). A two-tailed Student's t-test was used to determine the statistical significance among drug treatment groups. Each assay was repeated at least twice, and representative results were presented. *P* <0.05 was considered to be statistically significant. The IC50 value was calculated by Prism 5 (Graphpad Software Inc., La Jolla, CA).

## SUPPLEMENTARY FIGURES



## Abbreviations

NB: neuroblastoma
MDM2: Murine double minute 2 homolog
WT: wild type
Dox: doxorubicin
DMSO: dimethyl sulfoxide
FDA: Food and Drug Administration
RT-PCR: Reverse transcription polymerase chain reaction;

## References

[1] Maris JM (2010). Recent advances in neuroblastoma. The New England journal of medicine.

[2] Barbieri E, De Preter K, Capasso M, Johansson P, Man TK, Chen Z, Stowers P, Tonini GP, Speleman F, Shohet JM (2013). A p53 drug response signature identifies prognostic genes in high-risk neuroblastoma. PloS one.

[3] Donghi R, Longoni A, Pilotti S, Michieli P, Della Porta G, Pierotti MA (1993). Gene p53 mutations are restricted to poorly differentiated and undifferentiated carcinomas of the thyroid gland. The Journal of clinical investigation.

[4] Davidoff AM, Pence JC, Shorter NA, Iglehart JD, Marks JR (1992). Expression of p53 in human neuroblastoma- and neuroepithelioma-derived cell lines. Oncogene.

[5] Geoerger B, van Beusechem VW, Opolon P, Morizet J, Laudani L, Lecluse Y, Barrois M, Idema S, Grill J, Gerritsen WR, Vassal G (2005). Expression of p53, or targeting towards EGFR, enhances the oncolytic potency of conditionally replicative adenovirus against neuroblastoma. The journal of gene medicine.

[6] Goldschneider D, Blanc E, Raguenez G, Barrois M, Legrand A, Le Roux G, Haddada H, Benard J, Douc-Rasy S (2004). Differential response of p53 target genes to p73 overexpression in SH-SY5Y neuroblastoma cell line. Journal of cell science.

[7] He J, Gu L, Zhang H, Zhou M (2011). Crosstalk between MYCN and MDM2-p53 signal pathways regulates tumor cell growth and apoptosis in neuroblastoma. Cell cycle.

[8] Ago K, Shibutani M, Saegusa Y, Shima T, Taniai E, Mitsumori K (2009). A case report of a cerebellar neuroblastoma in a p53 null mutation mouse. The Journal of veterinary medical science / the Japanese Society of Veterinary Science.

[9] Barone G, Tweddle DA, Shohet JM, Chesler L, Moreno L, Pearson AD, Van Maerken T (2014). MDM2-p53 interaction in paediatric solid tumours: preclinical rationale, biomarkers and resistance. Current drug targets.

[10] Bu X, Shen Z, Jin B (1997). [Expression of p53 in nephroblastoma and neuroblastoma]. Zhonghua wai ke za zhi [Chinese journal of surgery].

[11] Burmakin M, Shi Y, Hedstrom E, Kogner P, Selivanova G (2013). Dual targeting of wild-type and mutant p53 by small molecule RITA results in the inhibition of N-Myc and key survival oncogenes and kills neuroblastoma cells in vivo and in vitro. Clinical cancer research.

[12] Chen L, Iraci N, Gherardi S, Gamble LD, Wood KM, Perini G, Lunec J, Tweddle DA (2010). p53 is a direct transcriptional target of MYCN in neuroblastoma. Cancer research.

[13] Choi MS, Yuk DY, Oh JH, Jung HY, Han SB, Moon DC, Hong JT (2008). Berberine inhibits human neuroblastoma cell growth through induction of p53-dependent apoptosis. Anticancer research.

[14] Condorelli F, Gnemmi I, Vallario A, Genazzani AA, Canonico PL (2008). Inhibitors of histone deacetylase (HDAC) restore the p53 pathway in neuroblastoma cells. British journal of pharmacology.

[15] Cui H, Schroering A, Ding HF (2002). p53 mediates DNA damaging drug-induced apoptosis through a caspase-9-dependent pathway in SH-SY5Y neuroblastoma cells. Molecular cancer therapeutics.

[16] Inomistova MV, Svergun NM, Khranovska NM, Skachkova OV, Gorbach OI, Klymnyuk GI (2015). Prognostic significance of MDM2 gene expression in childhood neuroblastoma. Exp Oncol.

[17] Nicolai S, Pieraccioli M, Peschiaroli A, Melino G, Raschella G (2015). Neuroblastoma: oncogenic mechanisms and therapeutic exploitation of necroptosis. Cell death & disease.

[18] Isaacs JS, Saito S, Neckers LM (2001). Requirement for HDM2 activity in the rapid degradation of p53 in neuroblastoma. The Journal of biological chemistry.

[19] Chen L, Tweddle DA (2012). p53, SKP2, and DKK3 as MYCN Target Genes and Their Potential Therapeutic Significance. Frontiers in oncology.

[20] Cheng J, Fan YH, Xu X, Zhang H, Dou J, Tang Y, Zhong X, Rojas Y, Yu Y, Zhao Y, Vasudevan SA, Zhang H, Nuchtern JG (2014). A small-molecule inhibitor of UBE2N induces neuroblastoma cell death via activation of p53 and JNK pathways. Cell Death Dis.

[21] Fan YH, Cheng J, Vasudevan SA, Dou J, Zhang H, Patel RH, Ma IT, Rojas Y, Zhao Y, Yu Y, Zhang H, Shohet JM, Nuchtern JG (2013). USP7 inhibitor P22077 inhibits neuroblastoma growth via inducing p53-mediated apoptosis. Cell Death Dis.

[22] Gamble LD, Kees UR, Tweddle DA, Lunec J (2012). MYCN sensitizes neuroblastoma to the MDM2-p53 antagonists Nutlin-3 and MI-63. Oncogene.

[23] Gillory LA, Stewart JE, Megison ML, Waters AM, Beierle EA (2015). Focal adhesion kinase and p53 synergistically decrease neuroblastoma cell survival. The Journal of surgical research.

[24] Wei JS, Song YK, Durinck S, Chen QR, Cheuk AT, Tsang P, Zhang Q, Thiele CJ, Slack A, Shohet J, Khan J (2008). The MYCN oncogene is a direct target of miR-34a. Oncogene.

[25] Slack A, Chen Z, Tonelli R, Pule M, Hunt L, Pession A, Shohet JM (2005). The p53 regulatory gene MDM2 is a direct transcriptional target of MYCN in neuroblastoma. Proc Natl Acad Sci U S A.

[26] Richter M, Dayaram T, Gilmartin AG, Ganji G, Pemmasani SK, Van Der Key H, Shohet JM, Donehower LA, Kumar R (2015). WIP1 phosphatase as a potential therapeutic target in neuroblastoma. PLoS One.

[27] Saito-Ohara F, Imoto I, Inoue J, Hosoi H, Nakagawara A, Sugimoto T, Inazawa J (2003). PPM1D is a potential target for 17q gain in neuroblastoma. Cancer Res.

[28] Shi Y, Ma IT, Patel RH, Shang X, Chen Z, Zhao Y, Cheng J, Fan Y, Rojas Y, Barbieri E, Chen Z, Yu Y, Jin J (2015). NSC-87877 inhibits DUSP26 function in neuroblastoma resulting in p53-mediated apoptosis. Cell Death Dis.

[29] Shang X, Vasudevan SA, Yu Y, Ge N, Ludwig AD, Wesson CL, Wang K, Burlingame SM, Zhao YJ, Rao PH, Lu X, Russell HV, Okcu MF (2010). Dual-specificity phosphatase 26 is a novel p53 phosphatase and inhibits p53 tumor suppressor functions in human neuroblastoma. Oncogene.

[30] Bill KL, Garnett J, Meaux I, Ma X, Creighton CJ, Bolshakov S, Barriere C, Debussche L, Lazar AJ, Prudner BC, Casadei L, Braggio D, Lopez G (2016). SAR405838: A Novel and Potent Inhibitor of the MDM2:p53 Axis for the Treatment of Dedifferentiated Liposarcoma. Clinical cancer research.

[31] Canner JA, Sobo M, Ball S, Hutzen B, DeAngelis S, Willis W, Studebaker AW, Ding K, Wang S, Yang D, Lin J (2009). MI-63: a novel small-molecule inhibitor targets MDM2 and induces apoptosis in embryonal and alveolar rhabdomyosarcoma cells with wild-type p53. British journal of cancer.

[32] Lau L, Hansford LM, Cheng LS, Hang M, Baruchel S, Kaplan DR, Irwin MS (2007). Cyclooxygenase inhibitors modulate the p53/HDM2 pathway and enhance chemotherapy-induced apoptosis in neuroblastoma. Oncogene.

[33] Michaelis M, Rothweiler F, Agha B, Barth S, Voges Y, Loschmann N, von Deimling A, Breitling R, Doerr HW, Rodel F, Speidel D, Cinatl J (2012). Human neuroblastoma cells with acquired resistance to the p53 activator RITA retain functional p53 and sensitivity to other p53 activating agents. Cell death & disease.

[34] Nag S, Qin J, Srivenugopal KS, Wang M, Zhang R (2013). The MDM2-p53 pathway revisited. Journal of biomedical research.

[35] Shangary S, Wang S (2009). Small-molecule inhibitors of the MDM2-p53 protein-protein interaction to reactivate p53 function: a novel approach for cancer therapy. Annual review of pharmacology and toxicology.

[36] Lakoma A, Barbieri E, Agarwal S, Jackson J, Chen Z, Kim Y, McVay M, Shohet JM, Kim ES (2015). The MDM2 small-molecule inhibitor RG7388 leads to potent tumor inhibition in p53 wild-type neuroblastoma. Cell death discovery.

[37] Hoffman-Luca CG, Yang CY, Lu J, Ziazadeh D, McEachern D, Debussche L, Wang S (2015). Significant Differences in the Development of Acquired Resistance to the MDM2 Inhibitor SAR405838 between In Vitro and In Vivo Drug Treatment. PloS one.

[38] Wang S, Sun W, Zhao Y, McEachern D, Meaux I, Barriere C, Stuckey JA, Meagher JL, Bai L, Liu L, Hoffman-Luca CG, Lu J, Shangary S, Yu S, Bernard D, Aguilar A (2014). SAR405838: an optimized inhibitor of MDM2-p53 interaction that induces complete and durable tumor regression. Cancer research.

[39] Meng X, Franklin DA, Dong J, Zhang Y (2014). MDM2-p53 pathway in hepatocellular carcinoma. Cancer research.

[40] Tucker ER, Danielson LS, Innocenti P, Chesler L (2015). Tackling Crizotinib Resistance: The Pathway from Drug Discovery to the Pediatric Clinic. Cancer research.

[41] Chen L, Rousseau RF, Middleton SA, Nichols GL, Newell DR, Lunec J, Tweddle DA (2015). Pre-clinical evaluation of the MDM2-p53 antagonist RG7388 alone and in combination with chemotherapy in neuroblastoma. Oncotarget.

[42] Deisenroth C, Zhang Y (2010). Ribosome biogenesis surveillance: probing the ribosomal protein-Mdm2-p53 pathway. Oncogene.

[43] Gangopadhyay S, Jalali F, Reda D, Peacock J, Bristow RG, Benchimol S (2002). Expression of different mutant p53 transgenes in neuroblastoma cells leads to different cellular responses to genotoxic agents. Experimental cell research.

[44] Wolter J, Angelini P, Irwin M (2010). p53 family: Therapeutic targets in neuroblastoma. Future oncology.

[45] Leontieva OV, Gudkov AV, Blagosklonny MV (2010). Weak p53 permits senescence during cell cycle arrest. Cell cycle.

[46] Zanjirband M, Edmondson RJ, Lunec J (2016). Pre-clinical efficacy and synergistic potential of the MDM2-p53 antagonists, Nutlin-3 and RG7388, as single agents and in combined treatment with cisplatin in ovarian cancer. Oncotarget.

[47] Deben C, Wouters A, Op de Beeck K, van Den Bossche J, Jacobs J, Zwaenepoel K, Peeters M, Van Meerbeeck J, Lardon F, Rolfo C, Deschoolmeester V, Pauwels P (2015). The MDM2-inhibitor Nutlin-3 synergizes with cisplatin to induce p53 dependent tumor cell apoptosis in non-small cell lung cancer. Oncotarget.

[48] Xu X, Hegazy WA, Guo L, Gao X, Courtney AN, Kurbanov S, Liu D, Tian G, Manuel ER, Diamond DJ, Hensel M, Metelitsa LS (2014). Effective cancer vaccine platform based on attenuated salmonella and a type III secretion system. Cancer research.

[49] Wang Y, Wang L, Guan S, Cao W, Wang H, Chen Z, Zhao Y, Yu Y, Zhang H, Pang JC, Huang SL, Akiyama Y, Yang Y (2016). Novel ALK inhibitor AZD3463 inhibits neuroblastoma growth by overcoming crizotinib resistance and inducing apoptosis. Scientific reports.

