# Supplementary material for: Novel MDM2 inhibitor SAR405838 (MI-773) induces p53-mediated apoptosis in neuroblastoma
Source: Oncotarget. 2016 Oct 13;7(50):82757–69. doi: 10.18632/oncotarget.12634 (PMC5347730; doi:10.18632/oncotarget.12634)
Supplement: Supplementary file 1 [file oncotarget-07-82757-s001.pdf]

Novel MDM2 inhibitor SAR405838 (MI-773) induces p53-mediated apoptosis in neuroblastoma

SUPPLEMENTARY FIGURES

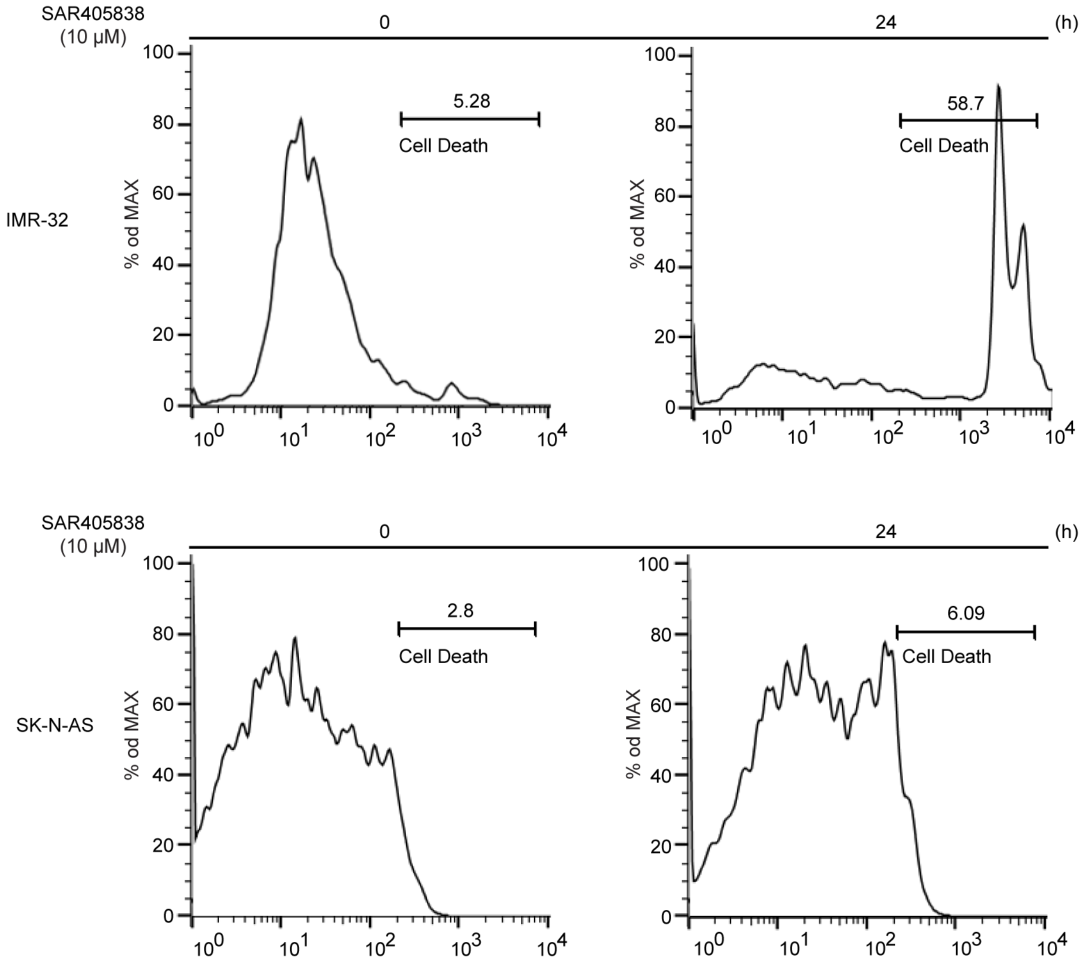

Supplementary Figure S1: SAR405838 induces cell death of p53 wild-type NB cell line IMR-32. The p53 wild-type IMR-32 and p53 mutant SK-N-AS cell lines were treated with 10 µM SAR405838 for 0 or 24 hours. The percentage of cell death in the treated cells was detected by flow cytometry analysis.

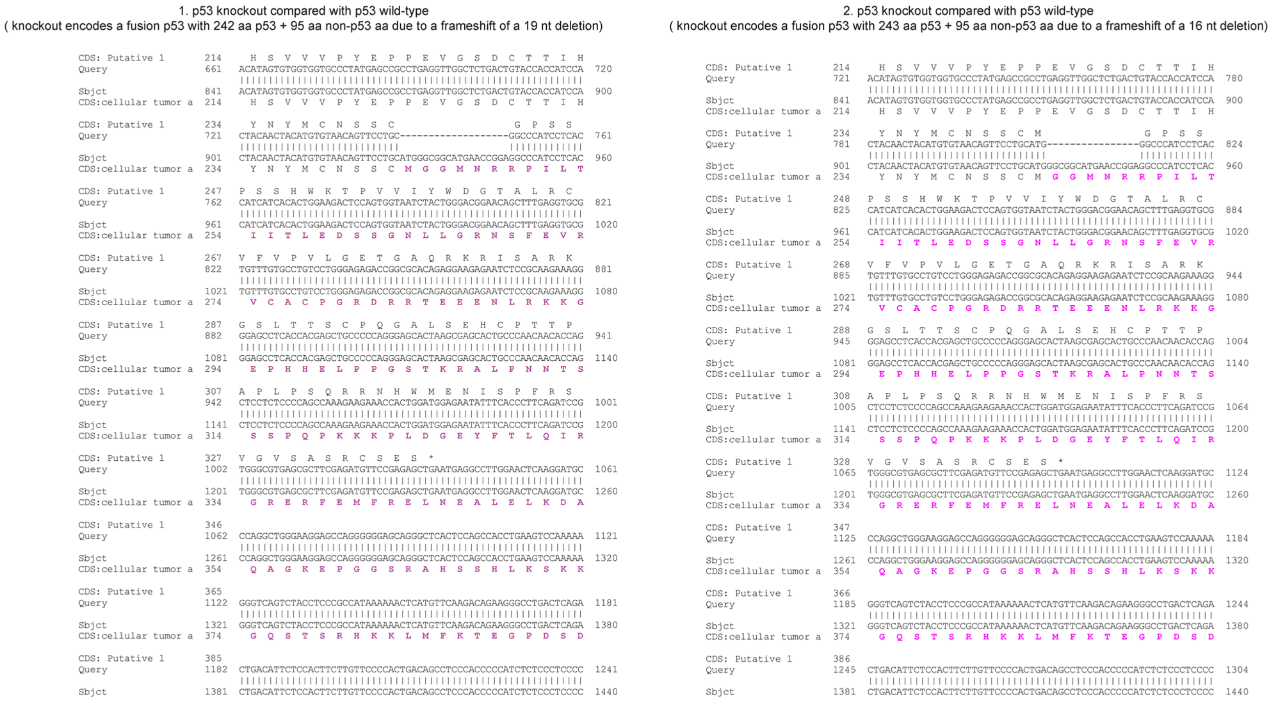

Supplementary Figure S2: The p53-knockout information in SH-SY5Y cells via the CRISPR-Cas9-mediated genomic editing technology. The lenti-viral vectors carrying the cassettes for a p53 gRNA and Cas9-2A-puromycin were transfected with packaging vectors into 293T cells for generating virus. The wild-type SH-SY5Y cells were transduced with lenti-virus for p53 knockout and then selected with puromycin. The single cell clone with p53 knockout was verified by PCR flanking the target site followed by cloning the PCR products and sequencing analysis.
